# Evaluation of thiamine and riboflavin in different varieties of rice grown in Kurigram district, Bangladesh

**DOI:** 10.1016/j.heliyon.2024.e39868

**Published:** 2024-10-26

**Authors:** Md Ashraf Ali, Md Zakir Sultan, Md Lemon Mia, Aminul Islam, Umme Habiba Ria, Md Abdus Salam

**Affiliations:** a Department of Chemistry, University of Dhaka, Dhaka, 1000, Bangladesh; b Centre for Advanced Research in Sciences (CARS), University of Dhaka, Dhaka, 1000, Bangladesh

**Keywords:** Thiamine, Riboflavin, HPLC, Fluorescence detector, Sunned rice, Parboiled rice

## Abstract

**Context:**

Rice is indeed a staple food in many parts of the world, including Bangladesh. This research aimed to evaluate the two essential vitamin levels in different processed rice sunned, parboiled, cooked sunned, cooked parboiled, and washed cooked rice.

**Objective:**

The study focused on analyzing the thiamine (vitamin B_1_) and riboflavin (vitamin B_2_) contents in 10 different varieties of paddy samples grown in the Kurigram district of Bangladesh.

**Methods:**

A high performance liquid chromatographic (HPLC) technique assembled with fluorescence detector was employed for these analyses. Two calibration curves were generated to analyze vitamin B_1_ and B_2_ (for thiamine the regression coefficient, r^2^ = 0.9992 and riboflavin r^2^ = 0.9989). The study demonstrated a recovery rate of 96.88 % for vitamin B_1_ and 104.24 % for vitamin B_2_. Overall, these findings provide valuable insights into the vitamin B_1_ and B_2_ contents across different rice samples.

**Results and conclusion:**

The results showed that the parboiled samples had the highest thiamine content, ranging from 342.50 ± 0.3 to 673.0 ± 7.5 μg/100 g, compared to the sunned samples, which had a range of 209.5 ± 0.3 to 337.4 ± 4.3 μg/100 g. Conversely, the sunned samples had the highest riboflavin content, ranging from 26.4 ± 0.2 to 59.6 ± 0.3 μg/100 g, while the parboiled samples had a range of 3.0 ± 0.1 to 26.6 ± 0.01 μg/100 g. Additionally, it was observed that the steaming process increased the thiamine content and reduced the riboflavin content in parboiled rice samples but decreased both the thiamine and riboflavin in sunned rice samples. Another study showed a gradual decrease in the levels of both vitamins B_1_ and B_2_ in sunned, cooked sunned and washed cooked sunned rice samples.

**Significance:**

These findings provide valuable insights into the impact of processing methods, such as parboiling and steaming, on the thiamine and riboflavin contents in rice. The results suggest that parboiled rice may be a preferable choice for individuals seeking higher thiamine content in their diet, while sunned rice may contain higher levels of riboflavin. The results shed light on the nutritional profiles of different rice varieties and highlight the potential impact of processing techniques on the vitamin content of rice.

## Highlights

• Thiamine and riboflavin content of different paddy grown in Kurigram were analyzed.
• They were sunned, parboiled, cooked sunned, cooked parboiled & washed cooked rice.
• Highest range of thiamine was found in parboiled & riboflavin in sunned samples.
• Steaming process increased thiamine & reduced riboflavin content in parboiled rice.
• Steaming process decreased both the thiamine & riboflavin for sunned rice samples.

## Graphical abstract

**Figure:**
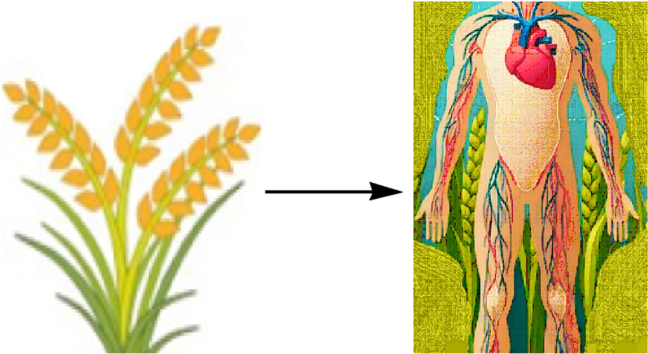

## 1 Introduction

Bangladesh is a nation in South Asia. With a population of almost 169 million people living in an area of 148,460 square kilometers, it is the eighth most populous country in the world [1]. Kurigram is a rural district, one of the 64 districts of Bangladesh, with an agrarian economy and a rich cultural heritage. It faces challenges related to agriculture, infrastructure, and natural disasters, but its people continue to work toward development and progress. This district in Bangladesh is highly vulnerable to crop intensification and enhancing crop production due to its unique land type and eco-geography [2]. A significant portion of the district consists of newly developed and established Char-lands, with 222 Chars formed by the flow of 16 rivers and their branches. These Chars cover nearly 23,000 ha of cultivated land and are primarily inhabited by marginalized and poor farmers. The formation of Chars is a result of siltation from upstream and erosion of rivers such as Tista, Dharla, Dudkumar, and Gangadhar [3]. The soil in these areas is predominantly sandy, with poor water-holding capacity and nutrient content. Almost every year, the Char-lands face prolonged inundation from floods or flash floods during the monsoon season. The frequency of flooding has been increasing, potentially due to climate change. While floodwaters usually recede within a week or two, they cause significant damage to standing crops [4]. Furthermore, the Char-lands are often situated far from towns, resulting in limited communication facilities and limited access to modern agricultural technologies. Despite the challenges posed by low water holding capacity and poor soil nutrients, farmers predominantly grow Boro rice, which requires substantial irrigation and increases production costs. Water scarcity also poses a threat to non-rice crop production. Consequently, a large area remains uncultivated or poorly cultivated during the dry season, resulting in low productivity. However, rice plays a crucial role in fulfilling this shortage and is highly relied upon as a staple food in the region. Therefore, it is essential to investigate whether this rice contains the necessary vitamins to meet nutritional requirements. To address this concern, a research study has been initiated to examine the presence of key vitamins, particularly vitamin B_1_ (thiamine) and B_2_ (riboflavin) in the rice. These two vitamins are analyzed by HPLC with different methods and techniques [[5], [6], [7]] but in this case, a high performance liquid chromatographic (HPLC) technique assembled with a fluorescence detector was employed for these analyses (see Table 1).

**Table 1:** Conditions for determination of Thiamine and Riboflavin.

Variable	For Thiamine	For Riboflavin
Column	Reversed phase C18 (4.6 mm × 250 mm, 5 μm)	
Mobile phase	0.005M hexane sulphonic acid in 85:15 methanol: water, pH 6.0	50 % methanol in water
Oxidant	0.001M potassium ferricyanide in 0.375 M sodium hydroxide	
Detector	Fluorescence, excitation 360 nm, emission 435 nm	Fluorescence, excitation 440 nm, emission 530 nm
Injection Volume	10 μL	10 μL
Flow Rate	1.0 mL/min (both the mobile phase and the oxidant)	1.0 mL/min
Run Time	10 min	
Gradient	Isocratic	

This research aims to evaluate the nutritional composition of the rice samples collected from the Kurigram district and assess the levels of vitamins B_1_ and B_2_. By conducting this study, the potential contribution of rice in meeting the nutritional needs of the local population gain insights. The findings will provide valuable information for policymakers, healthcare professionals, and the community to make informed decisions regarding food security and public health initiatives.

Thiamine and riboflavin are both essential vitamins for human health. Both of these improve the immune system and are essential for energy metabolism, nervous system function, cardiovascular health, skin health, eye health, antioxidant activity, red blood cell production, brain function, digestive health, stress management, etc. Therefore, the objective of the study is the evaluation of thiamine and riboflavin in rice varieties to ensure the benefit of health.

## 2 Materials and methods

**Study area:** The different varieties of rice samples were collected from the district Kurigram, Bangladesh and analyses were carried out in the Centre for Advanced Research in Sciences, University of Dhaka, Dhaka-1000, Bangladesh in 2023.

**Materials:** All the chemicals and solvents used in this study are methanol, 1-hexanesulfonate sodium salt, sodium hydrogencarbonate salt, hydrochloric acid, sodium acetate, takadiastase, potassium ferricyanide, sodium hydroxide, ethanoic acid, ethanol [All the chemicals were supplied by Sigma Aldrich, India], distilled water, thiamine hydrochloride (vitamin B1), and riboflavin (vitamin B2) [Bellefonte, PA, USA]. An HPLC (Shimadzu, Japan) assembled with are reversed-phase C-18 column [C-18 Borosil], a fluorescence detector [Shimazdu, Japan] was used. Vacuum pump filter [Sigma Aldrich, India], heating oven [Memmert, ThermoFisher Scientific, USA], electronic balance [Radwag, UK], ultra-sonicator [J.P. Selecta, Spain], micropipette [BOECO, Germany], water distillation plant [IndiaMart, India], syringe filter [Restek, France], spatula and needle [JMI group, Bangladesh], mixing block (T-piece) [Shimazdu, Japan], ultrasonic water bath [J.P. Selecta, Spain], vortex mixture [Digisystem, Taiwan], pH meter were also used for qualitative and quantitative analysis.

## 3 Methodology

In this research, a total of nineteen (19) rice samples of ten (10) paddy varieties were analyzed. Out of these, eighteen (18) samples were collected from the rural area of Phulbari Upazila in Kurigram District, Bangladesh, while one (01) sample was obtained from a local area in Dhaka City, Bangladesh. Among the eighteen (18) samples, they were all husking rice that had been processed by using a husking machine (Manufactured by Bangladesh). The remaining one (01) sample was polishing rice. Further categorizing the samples, ten (10) of them were sunned rice, while the other nine (9) samples were parboiled rice. This diverse set of samples allowed us to explore and compare different types of rice commonly consumed at Kurigram Zilla in Bangladesh. The scientific name of rice/paddy is *oryza sativa*. The name of the paddy varieties were BRRI Dhan 29 [Bangladesh, BRRI], Janakraj SQR-6 [China, National AgriCare], SL – 8H [Philippines, BADC], BRRI Dhan28 [Bangladesh, BRRI], Krishibid Farm Hybrid Dhan-1 [China, Krishibid Farm Limited], Super 28 (Hybrid) [Bangladesh, BRRI], Swarna (MTU-7029) [India, IRRI], BR-09 (Sufala) [Bangladesh, BRRI], BR 34 (Chinigura) [Bangladesh, BRRI], BRRI hybrid Dhan-2 [Sunned rice] [Bangladesh, BRRI]. Approximately 2 kg of each type of rice was collected. Of this, 1 kg was boiled with about 200 mL of water for around 15 min and then sun-dried [Parboiled rice]. The remaining 1 kg was directly sun-dried [Sunned rice]. This method followed traditional regional practices for parboiling rice. Finally, the outer husk of the rice was removed using a traditional wooden mortar and pestle. Additionally, approximately 10 g of powdered rice was cooked with 50 mL of water at 70 °C to prepare cooked rice [Cooked rice].

### 3.1 Extraction processes of thiamine and riboflavin from the rice samples

To enhance the extraction of thiamine and riboflavin from rice samples for subsequent HPLC analyses, a total of approximately 30g of each rice sample was carefully powdered using a laboratory-grade grinder to ensure uniformity. The samples were ground until a consistent particle size was achieved. The average particle size of the powdered samples was approximately 100–200 μm, which is a standard range for powdered rice in Bangladesh. This meticulous process was employed to increase the surface area of the rice particles and facilitate greater interaction between the sample and the extraction solvent, thus the utilization of powdered rice samples in this study was a deliberate and strategic choice, aimed at maximizing the extraction efficiency of thiamine and riboflavin. This methodology contributes to the robustness of the overall analytical procedure, ensuring the accurate determination of these important vitamins in rice samples. About 10 g of each powdered rice sample was accurately weighed by using an electronic balance in a 250 mL conical flask. About 100 mL of 0.1 M hydrochloric acid (HCl) was added to all flasks then capped with aluminum foil and mixed. The flasks were placed in an ultrasonic boiling water bath for 30 min. Then the flasks were removed from the ultrasonic water bath and then cooled below 50 °C. The flasks were added about 8 mL of 10 % Takadiastase solution and then capped the flasks, mixed, and further incubated in a 37 °C ultrasonic water bath overnight. These were cooled to room temperature and filtered through the filter paper (Whatman® No. 42). Finally, the filtrate was collected into a 150 mL volumetric flask (a 100 mL and a 50 mL volumetric flask). Then the filtrate was filtered further through a 0.45 μm filter unit and a 1.5 mL amber glass vial was filled with each of the solutions which was ready for HPLC analysis. The same extraction process was followed to extract five different concentrations of composite intermediate of thiamine and riboflavin [[8], [9], [10], [11], [12]] and rice sample.

### 3.2 HPLC analyses

The vitamins were analyzed by a HPLC assembled with a fluorescence detector by using the method described as follows-

∗In HPLC analysis, thiamine is determined under oxidation conditions using 0.001 M potassium ferricyanide in 0.375 M sodium hydroxide. Potassium ferricyanide acts as a strong oxidizing agent, converting thiamine into its oxidized form, which enhances the accuracy and precision of its detection and quantification. Sodium hydroxide, being a strong base, maintains the pH and stability of the solution during the analysis. The mobile phase was passed through pump-B within the HPLC column whereas an oxidant was introduced at the T-piece, which was placed between the column and the detector. On the other hand, riboflavin is determined under normal conditions without the need for oxidation or reduction.

### 3.3 Statistical analysis

Statistical analysis in the context of this study involves using both Microsoft Excel and SPSS. Microsoft Excel's built-in functions and tools were utilized to calculate and interpret the initial data set. This process includes drawing insights, summarizing key features through descriptive statistics, conducting inferential statistics for hypothesis testing, and creating visual representations of data using charts and graphs. Excel's capabilities extend to correlation, regression analysis, and efficient data cleaning and preprocessing tasks, making it a versatile tool for basic statistical analysis. To validate our findings, we performed a one-way ANOVA using SPSS. The one-way ANOVA was chosen to evaluate the differences among the groups, and it provided a deeper level of statistical rigor. The results from the ANOVA revealed that there were significant differences between the groups (*p* < 0.05), indicating that the treatment had a notable effect on the outcomes. These results are presented and discussed in the Results section.

## 4 Results

**Fig. 1:**
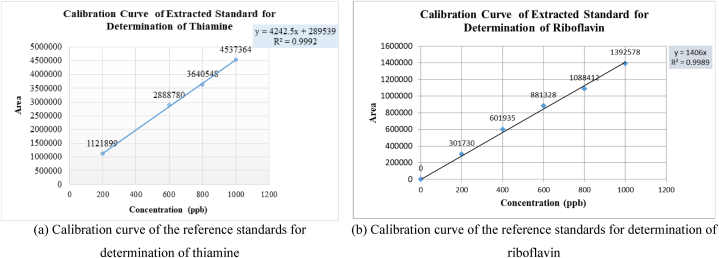
Calibration curves of the reference standards for determination of (a) thiamine, and (b) riboflavin for all rice samples.

Five different concentration levels of 200, 400, 600, 800, and 1000 ppb were prepared from the standard composite intermediate of thiamine and riboflavin. Then 10 μL from each solution was injected into the HPLC using an auto-sampler and the analyses were monitored by a fluorescence detector. Then the peak areas were plotted against concentrations. Two calibration curves were generated to estimate the quantities of thiamine and riboflavin. These curves were shown in Fig. 1.

**Fig. 2:**
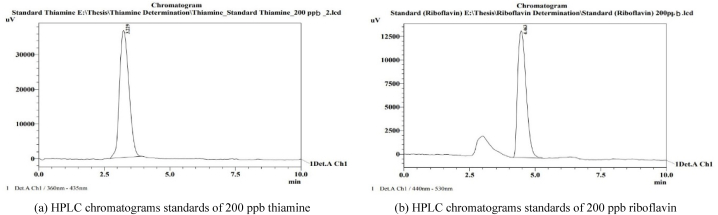
HPLC chromatograms (a) standards of 200 ppb thiamine, (b) standards of 200 ppb riboflavin.

Two HPLC chromatograms obtained from extracted standards of 200 ppb, one for thiamine, monitored by a fluorescence detector at the 360 nm excitation wavelength and the 435 nm emission wavelength and another one for riboflavin, monitored by a fluorescence detector at the 440 nm excitation wavelength and the 530 nm emission wavelength, are shown Fig. 2.

**Fig. 3:**
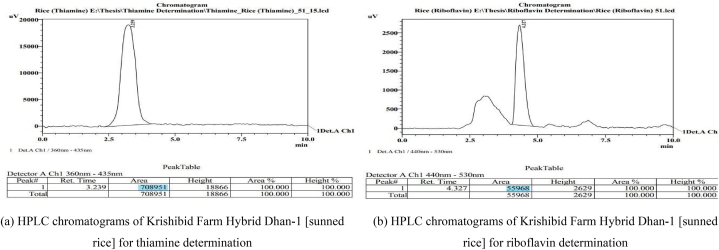
HPLC chromatograms of Krishibid Farm Hybrid Dhan-1 [sunned rice] rice sample: (a) for thiamine determination, (b) for riboflavin determination.

**Chromatograms of rice samples:** Two examples of chromatograms of rice samples are provided in Fig. 3: one for thiamine and one for riboflavin, both obtained under the same analysis conditions as the standard thiamine and riboflavin.

### 4.1 Analyses data

The data in the table were derived by averaging the results from three experiments, expressed as Mean ± Standard Error of the Mean (SEM). Statistical analysis was performed using one-way ANOVA in SPSS at a 95 % confidence level, and statistical significance was accepted at *p* < 0.05. The ∗ symbol in superscript signifies that the data is statistically significant.

**Fig. 4:**
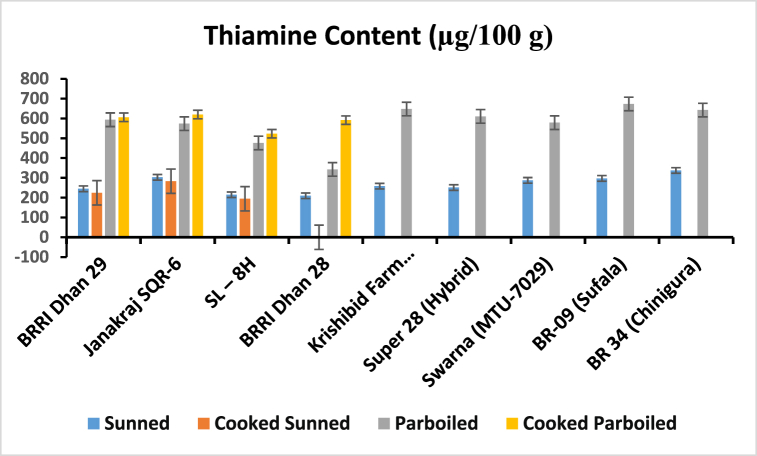
Comparison of the thiamine in Sunned, Parboiled, Cooked Sunned, and Cooked Parboiled Rice.

**Fig. 5:**
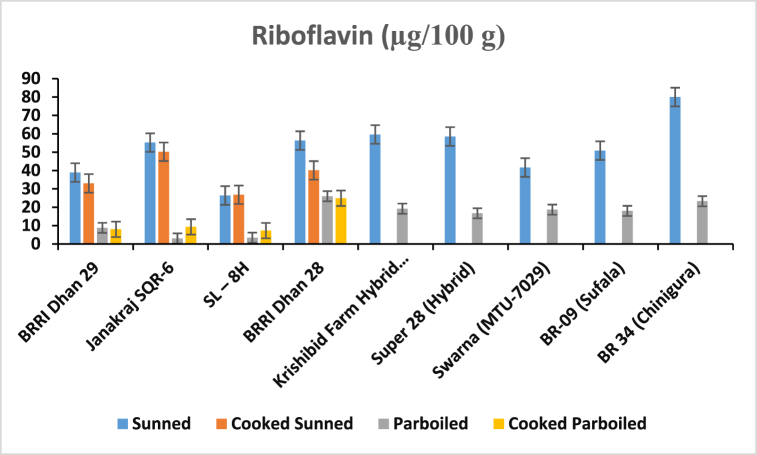
Comparison of riboflavin in sunned, parboiled, cooked sunned, and cooked parboiled rice.

After analyzing the samples by HPLC, the contents of thiamine and riboflavin were calculated which are mentioned in Fig. 4, Fig. 5. This information will help us assess the nutritional profiles of Sunned, Cooked Sunned, Parboiled, and Cooked Parboiled Rice and understand any differences that may exist among them in terms of these essential B vitamins. The comparison of the amount of thiamine and riboflavin (μg/100 g) in different rice samples is given (see Table 2).

**Fig. 6:**
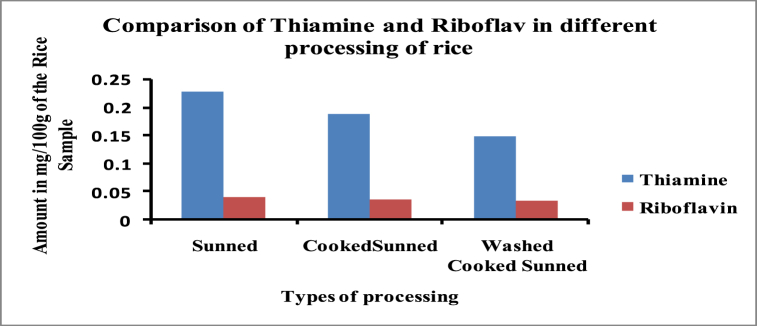
Comparison of thiamine and riboflavin in sunned, cooked sunned, and washed cooked sunned rice.

The comparison of the amount of thiamine and riboflavin (μg/100 g) in only one rice variety but in its sunned, cooked sunned, and washed cooked sunned rice is given in Table 3 and Fig. 6.

**Table 2:** Comparison of thiamine and riboflavin in Sunned, Cooked Sunned, Parboiled, and Cooked Parboiled Rice.

Serial No.	Variety Name	Thiamine (μg/100 g)				Riboflavin (μg/100 g}			
		Sunned	Cooked Sunned	Parboiled	Cooked Parboiled	Sunned	Cooked Sunned	Parboiled	Cooked Parboiled
1.	BRRI Dhan 29	245.0 ± 2.9	224.7 ± 0.4	593.4 ± 2.0∗	605.7 ± 3.3∗	38.9 ± 0.5	33.0 ± 0.6	8.8 ± 0.1	8.0 ± 0.3
2.	Janakraj SQR-6	302.8 ± 1.6	283.3 ± 1.9	573.8 ± 2.2∗	619.6 ± 0.2∗	55.2 ± 0.1∗	50.2 ± 0.1∗	3.0 ± 0.1	9.3 ± 0.1
3.	SL – 8H	214.8 ± 0.5	194.4 ± 0.2	476.0 ± 0.6∗	522.8 ± 0.5∗	26.4 ± 0.2	26.8 ± 0.2	3.4 ± 0.1	7.3 ± 0.1
4.	BRRI Dhan 28	209.5 ± 0.3	207.5 ± 0.6	342.5 ± 0.3∗	591.4 ± 0.8∗	56.3 ± 0.2∗	40.1 ± 0.3∗	26 ± 0.1∗	24.9 ± 0.2∗
5.	Krishibid Farm Hybrid Dhan-1	258.1 ± 4.7		647.7 ± 1.0		59.6 ± 0.3∗		19.2 ± 0.1∗	
6.	Super 28 (Hybrid)	251.1 ± 0.6		610.3 ± 5.9∗		58.5 ± 0.3∗		16.7 ± 0.2∗	
7.	Swarna (MTU-7029)	287.4 ± 1.5		578.7 ± 5.0∗		41.6 ± 0.3		18.7 ± 0.2∗	
8.	BR-09 (Sufala)	297.2 ± 4.2		673.0 ± 7.5∗		50.8 ± 0.3∗		18.0 ± 0.3∗	
9.	BR 34 (Chinigura)	337.4 ± 4.3∗		642.3 ± 1.3∗		80.0 ± 2.9∗		23.3 ± 0.2∗

**Table 3:** Comparison of thiamine and riboflavin in sunned, cooked sunned, and washed cooked sunned rice.

Serial No.	Vitamin Name	Amount in μg/100 g of the Rice Sample		
		Sunned	Cooked Sunned	Washed Cooked Sunned
1.	Thiamine	228.4	188.4	147.3
2.	Riboflavin	39.4	36.0	32.9

The recovery study calculation involved calculating the percentage of analyte recovery for the purpose to assess the level of accuracy of a specific analytical method. The objective of this study was to ensure an accurate measurement of the target compound and to confirm the method's dependability. To check the validity of the method, recovery study was performed based on %recovery at 5 replicates analyses for each vitamin (Table 4).

**Table 4:** Recovery study of thiamine and riboflavin.

Spiked Concentration (ppb)	Un-spiked Concentration (ppb)	Added standard concentration (ppb)	%Recovery
Recovery Study for Thiamine			
439.69	139.69	300	96.88
Recovery Study for Riboflavin			
417.76	17.76	400	104.24

## 5 Discussion

The study focused on evaluating the thiamine and riboflavin levels in different processed rice sunned, parboiled, cooked and washed cooking rice. High Performance Liquid Chromatographic (HPLC) technique was employed for the analyses. The regression coefficient of these analyses was 0.9992 for the calibration curve to determine thiamine content, and 0.9989 for the calibration curve to determine riboflavin content. The study demonstrates a recovery rate of 96.88 % for thiamine and 104.24 % for riboflavin, indicating the accuracy and reliability of the analysis method employed (Table 4). Now, let's focus on the ranges of thiamine content found in different varieties of rice samples (Fig. 4). For the sunned rice, the thiamine content ranged from 209.5 ± 0.3 to 337.4 ± 4.3 μg/100 g. In the case of cooked sunned rice, the range was slightly lower, ranging from 207.5 ± 0.6 to 283.3 ± 1.9 μg/100 g. Moving on to parboiled rice, the thiamine content exhibited a wider range, spanning from 342.5 ± 0.3 to 673.0 ± 7.5 μg/100 g. Lastly, the cooked parboiled rice had a narrower range, with thiamine content ranging from 522.8 ± 0.5 to 619.6 ± 0.2 μg/100 g. Now, let's shift attention to the ranges of riboflavin content in the various samples (Fig. 5). The sunned rice showed a range of 26.40 ± 0.2 to 59.60 ± 0.3 μg/100 g, whereas the cooked sunned rice had a slightly narrower range of 26.8 ± 0.2 to 50.2 ± 0.1 μg/100 g. Surprisingly, the parboiled samples had a significantly lower range, ranging from 3.0 ± 0.1 to 26.60 ± 0.1 μg/100 g. Lastly, the cooked parboiled rice exhibited an even narrower range, with riboflavin content ranging from 7.3 ± 0.1 to 24.9 ± 0.2 μg/100 g. The results show that the parboiled samples had the highest range of thiamine content, while the sunned samples had the highest range of Riboflavin content. In a study conducted by Padua et al. (1974), the effect of parboiling on the thiamine content of rice was examined. The findings revealed that parboiling led to a decrease in thiamine content [10]. A study by Kumari et al. (2019) examined the effect of parboiling and milling processes on thiamine retention in rice. They found that parboiling rice led to a significant reduction in thiamine content compared to raw rice, while milling had a minimal effect. The authors suggested that optimizing parboiling conditions could help minimize thiamine loss during processing [13]. Additionally, it was observed that the cooking process further increased the thiamine content and reduced the riboflavin content in parboiled rice samples but decreased both the thiamine and riboflavin for sunned rice samples. Another result showed in Fig. 6, a gradual decrease in the levels of both thiamine and riboflavin in sunned, cooked sunned, and washed cooked sunned rice samples. These results provide valuable insights into the impact of processing methods on the nutritional composition of rice, specifically regarding thiamine and riboflavin. It's important to consider these factors when evaluating the nutritional value of rice and when determining appropriate processing methods for rice production. A study by Zhang et al.*,* (2018) investigated the impact of nitrogen fertilizer application on thiamine accumulation in rice grains. They found that increased nitrogen fertilizer levels resulted in higher thiamine concentrations in rice grains. However, excessive nitrogen fertilization beyond optimal levels had a negative effect on thiamine content [14]. A study by Thavarajah et al. (2012) investigated the impact of parboiling and polishing on riboflavin retention in rice. They found that parboiled rice had higher riboflavin content compared to raw rice, while polishing resulted in a significant reduction in riboflavin levels. The authors suggested that minimizing polishing during rice processing could help retain riboflavin and improve its bioavailability [15]. A study by De Rosa et al. (2018) investigated the effect of nitrogen fertilization on riboflavin levels in rice plants. They observed that increasing nitrogen fertilizer levels resulted in higher riboflavin concentrations in rice grains. However, excessive nitrogen fertilization had negative impact on riboflavin content. This finding highlights the importance of optimizing fertilization practices to enhance riboflavin accumulation in rice [16].

## 6 Conclusion

The results of the study indicated that parboiled rice had approximately double the thiamine content compared to sunned rice. However, the riboflavin content in parboiled rice was found to be approximately half or greater than half that of sunned rice. This suggests that the parboiling process has both positive and negative effects on the B-vitamin content of rice. Additionally, the cooking process further increased the thiamine content and reduced the riboflavin content in parboiled rice samples but decreased both the thiamine and riboflavin for sunned rice samples. This indicates that cooking has a detrimental impact on the retention of these B vitamins during rice processing. Another study showed a gradual decrease in the levels of both thiamine and riboflavin in sunned, cooked sunned, and washed cooked sunned rice samples. This suggests that the overall processing and preparation methods employed on rice can lead to a decline in the levels of these essential B vitamins. In conclusion, thiamine and riboflavin are both water-soluble vitamins that are sensitive to environmental conditions. When rice is exposed to sunlight or heat during sunning or cooking processes, it can lead to the degradation of these vitamins over time. During cooking, the rice is exposed to heat and moisture, which can cause minimal loss of thiamine. However, the overall thiamine content may still be higher in cooked parboiled rice compared to raw rice due to the redistribution of thiamine during the parboiling process. Additionally, the presence of water during washing and cooking can further contribute to the loss of riboflavin. Light, especially ultraviolet (UV) light, can cause the breakdown of vitamins, including thiamine and riboflavin. Prolonged exposure to sunlight can accelerate the degradation process, resulting in reduced vitamin levels in the rice. The study recommends that consumers choose parboiled rice to maintain higher thiamine levels, as it retains more thiamine compared to other processed rice types. For higher riboflavin intake, consumers should opt for sunned rice, as the drying process preserves riboflavin better. Reducing washing can also help limit the loss of water-soluble vitamins like thiamine and riboflavin. For producers, the study suggests optimizing parboiling techniques to retain thiamine, promoting sun-drying methods to preserve riboflavin, and ensuring proper storage conditions (low humidity, minimal light exposure) to prevent vitamin degradation. Finally, the data indicates that parboiling and cooking parboiled rice typically result in higher thiamine and riboflavin content compared to sunned and cooked sunned rice. Specifically, BRRI Dhan 28 and Krishibid Farm Hybrid Dhan-1 (cooked parboiled) showed notably elevated thiamine levels, while BR 34 (Chinigura) also has high thiamine content, particularly in its sunned form. For riboflavin, parboiling and cooking parboiled rice generally enhance its levels across most varieties. BRRI Dhan 28 and Janakraj SQR-6 (cooked parboiled) exhibited significantly higher riboflavin content, and BR 34 (Chinigura) also demonstrated high riboflavin levels, especially in its sunned form. Overall, the processes of parboiling and cooking parboiled rice contributed to increase thiamine and riboflavin contents, with BRRI Dhan 28 and BR 34 (Chinigura) standing out for their high vitamin levels.

**Significance statement:** Thiamine and riboflavin play pivotal roles in a nutrient-rich environment, impacting essential physiological functions. Thiamine is crucial for energy metabolism, nerve function, and nucleic acid synthesis, while riboflavin is vital for metabolism, skin, eye health, and nerve cells. In a diet rich in these vitamins, individuals may experience heightened energy production, improved cognitive function, and overall well-being. Moreover, deficiencies in thiamine and riboflavin can lead to serious health issues, such as beriberi and neurological disorders. Recognizing the significance of these vitamins in a nutrient-rich context underscores the importance of targeted nutritional strategies to prevent deficiencies and promote optimal health. Future research should explore the long-term effects and potential synergies of these vitamins in diverse populations, paving the way for refined nutritional guidelines and personalized health interventions. The study found that both thiamine and riboflavin contents in rice decreased due to processing methods. Several factors contribute to this reduction, including: milling and polishing processes remove the outer layers of rice grains, which are rich in vitamins. The bran and germ, where thiamine and riboflavin are concentrated, are often lost during these processes. Milling can reduce thiamine by up to 85 % and riboflavin by up to 70 % [17]. Heat treatment methods, such as parboiling and steaming, can degrade heat-sensitive vitamins like thiamine and riboflavin. High temperatures and prolonged exposure to heat can cause these vitamins to break down [18]. Soaking and washing rice before cooking can lead to the leaching of water-soluble vitamins such as thiamine and riboflavin into the water, resulting in significant vitamin loss if the soaking water is discarded [19]. Improper storage conditions, such as high humidity and exposure to light, can also contribute to the degradation of vitamins in rice. Both thiamine and riboflavin are sensitive to environmental factors, leading to their gradual decline during storage [20,21]. Keeping the temperature consistent and within an optimal range prevents degradation. Controlling humidity levels prevents moisture-related damage. Dry environments are often preferable to avoid mold and mildew. Limiting exposure to light, especially UV light, helps in preserving both B1 and B2, as light can cause chemical breakdown. Using airtight and opaque packaging can protect B1 and B2 from environmental factors and contamination. One other thing, simultaneous determination of thiamine (vitamin B1) and riboflavin (vitamin B2) using HPLC is challenging due to their differing optimal detection conditions. Thiamine produces a strong chromatographic peak when oxidized to thiochrome, necessitating the use of an oxidizing agent such as potassium ferricyanide. In contrast, riboflavin provides well-defined peaks under normal, non-oxidizing conditions, where it naturally fluoresces. This discrepancy in required conditions means that a single HPLC method typically cannot accurately measure both vitamins simultaneously. Potential strategies to address this include sequential analysis under different conditions or developing novel derivatization techniques that stabilize both compounds for simultaneous detection. Despite these challenges, ongoing research aims to refine and optimize HPLC methods to achieve reliable and efficient simultaneous quantification of thiamine and riboflavin in various matrices.

## CRediT authorship contribution statement

**Md Ashraf Ali:** Writing – original draft, Validation, Resources, Methodology, Investigation, Formal analysis, Data curation. **Md Zakir Sultan:** Writing – review & editing, Visualization, Supervision, Resources, Project administration, Methodology, Investigation, Conceptualization. **Md Lemon Mia:** Validation, Resources, Investigation, Formal analysis, Data curation. **Aminul Islam:** Resources, Investigation, Formal analysis, Data curation. **Umme Habiba Ria:** Data curation. **Md Abdus Salam:** Supervision, Project administration.

## Data and code availability

Data will be made available on request.

## Declaration of competing interest

The authors declare that they have no known competing financial interests or personal relationships that could have appeared to influence the work reported in this paper.

## References

[1] Central Intelligence Agency. Bangladesh (2023). https://www.cia.gov/the-world-factbook/about/archives/2021/countries/bangladesh/.

[2] Islam M.A., Das P.R., Mahmud A.F.M.S.B., Hasan M.E., Jahan F.I., Seraj S., Islam F., Khatun Z., Chowdhury A.R., Rahman M.A., Rahmatullah M. (2011). A survey of non-conventional plant items consumed during food scarcity in two randomly selected villages of Kurigram district, Bangladesh. Am.-Eurasian J. Sustain. Agric. (AEJSA).

[3] Pal P.K., Rahman A., Yunus A. (2017). Analysis on river bank erosion-accretion and bar dynamics using multi-temporal satellite images. American Journal of Water Resources.

[4] Islam M.R., Begum S.F., Yamaguchi Y., Ogawa K. (1999). The Ganges and Brahmaputra rivers in Bangladesh: basin denudation and sedimentation. Hydrological Response.

[5] Horwitz W. (2000). Official methods of analysis of AOAC international. Thiamin Fluorometric Methods.

[6] Wehling R.L., Wetzel D.L. (1984). Simultaneous determination of pyridoxine, riboflavin, and thiamin in fortified products by high-performance liquid chromatography. J. Agric. Food Chem..

[7] Wimalasiri P., Wills R.B.H. (1985). Simultaneous analysis of thiamin and riboflavin in foods by high-performance liquid chromatography. Journal of Chromatography.

[8] Ellefson W.C., Augustin J., Klein B.P., Becker D.A., Venugopal P.B. (1985). Methods of Vitamin Assay.

[9] Shah J.J., Augustin J., Klein B.P., Becker D.A., Venugopal P.B. (1985). Methods of Vitamin Assay.

[10] Verma D.K. (2015). Physico-chemical and cooking characteristics of Azad basmati. Int. Food Res. J..

[11] Otegbayo B.O., Osamuel F., Fashakin J.B. (2001). Effect of parboiling on physico-chemical qualities of two local rice varieties in Nigeria. J. Food Technol. Afr..

[12] Mia M.L., Sultan M.Z., Ali M.A., Simol H.A., Salam M.A. (2024). Quantification of riboflavin and thiamine in GI (geographical indication) branded yogurts collected from bogura, Bangladesh using HPLC equipped with a fluorescence detector. Bangladesh Pharmaceutical Journal.

[13] Xie L., Li M., Xu L. (2017). Determination of thiamine contents in different rice varieties by high-performance liquid chromatography. Food Chem..

[14] Zhang Z., Yuan L., Chen F. (2018). Effect of nitrogen fertilizer on thiamine accumulation in rice grains. Journal of Central Science.

[15] Thavarajah D., Thavarajah P., Wejesuriya A. (2012). Riboflavin content of rice and its contribution to dietary intake. Food Chem..

[16] De Rosa M., Prisa D., Filippone E. (2018). Nitrogen fertilization influences riboflavin content and GTP cyclohydrolase II activity in rice grains. J. Agric. Food Chem..

[17] Steiger G., Müller-Fischer N., Cori H., Conde-Petit B. (2014). Fortification of rice: technologies and nutrients. Ann. N. Y. Acad. Sci..

[18] Khokhar S., Apenten R.K. (2003). Iron-binding characteristics of phenolic compounds: some tentative structure-activity relations. Food Chem..

[19] Chaudhary R.C. (2003). Speciality rices of the world: effect of WTO and IPR on its production trend and marketing. J. Food Agric. Environ..

[20] Chen M.H., Bergman C.J. (2008). Pasting properties in an international rice germplasm collection. J. Cereal. Sci..

[21] Fan C.C., Yu X.Q., Xing Y.Z., Xu C.G., Luo L.J., Zhang Q. (2005). The main effects, epistatic effects and environmental interactions of QTLs on the cooking and eating quality of rice in a double haploid line population. Theoretical and Applied Genetics.

